# Negative enrichment of circulating tumor cells from unmanipulated whole blood with a 3D printed device

**DOI:** 10.1038/s41598-021-99951-0

**Published:** 2021-10-18

**Authors:** Chia-Heng Chu, Ruxiu Liu, Tevhide Ozkaya-Ahmadov, Brandi E. Swain, Mert Boya, Bassel El-Rayes, Mehmet Akce, Mehmet Asim Bilen, Omer Kucuk, A. Fatih Sarioglu

**Affiliations:** 1 School of Electrical and Computer Engineering, Georgia Institute of Technology, Atlanta, USA; 2 Winship Cancer Institute, Emory University, Atlanta, USA; 3 Department of Hematology and Medical Oncology, Emory University School of Medicine, Atlanta, USA; 4 Parker H. Petit Institute for Bioengineering and Bioscience, Georgia Institute of Technology, Atlanta, USA; 5 Institute for Electronics and Nanotechnology, Georgia Institute of Technology, Atlanta, USA

**Keywords:** Lab-on-a-chip, Cancer screening, Cancer

## Abstract

Reliable and routine isolation of circulating tumor cells (CTCs) from peripheral blood would allow effective monitoring of the disease and guide the development of personalized treatments. Negative enrichment of CTCs by depleting normal blood cells ensures against a biased selection of a subpopulation and allows the assay to be applied on different tumor types. Here, we report an additively manufactured microfluidic device that can negatively enrich viable CTCs from clinically-relevant volumes of unmanipulated whole blood samples. Our device depletes nucleated blood cells based on their surface antigens and the smaller anucleated cells based on their size. Enriched CTCs are made available off the device in suspension making our technique compatible with standard immunocytochemical, molecular and functional assays. Our device could achieve a ~ 2.34-log depletion by capturing > 99.5% of white blood cells from 10 mL of whole blood while recovering > 90% of spiked tumor cells. Furthermore, we demonstrated the capability of the device to isolate CTCs from blood samples collected from patients (n = 15) with prostate and pancreatic cancers in a pilot study. A universal CTC assay that can differentiate tumor cells from normal blood cells with the specificity of clinically established membrane antigens yet require no label has the potential to enable routine blood-based tumor biopsies at the point-of-care.

## Introduction

Circulating tumor cells (CTC) hold the promise for non-surgical, minimally invasive tumor biopsies as well as personalized drug therapies optimized on patients’ tumor cells^1–5^, yet their practical isolation from peripheral blood remains a challenge. Not only are CTCs extremely rare among normal blood cells even in patients with metastatic disease^6,7^, but they also show differences among themselves in terms of chemical or physical appearance, making their successful detection from patient samples dependent on sensitive tools and specific biomarkers^8,9^.

CTCs are targeted in blood samples of cancer patients using a variety of technologies. Among these technologies, laboratory assays for CTC discrimination utilize different enrichment strategies, including size-based enrichment through filtration^10–12^, density gradient centrifugation^13^, optical and image-based detection^14,15^, as well as magnetic assisted cell sorting^16,17^. In addition to these conventional batch processes, microfluidic CTC detection approaches allow deterministic interrogation of individual blood cells through precisely-manufactured features and can often utilize localized force fields to achieve higher sensitivity and specificity^18–20^. To discriminate CTCs from blood cells, microfluidic devices were designed to exploit the biophysical and biochemical contrast between tumor and normal blood cells, with some simultaneously utilizing a combination of physical and chemical modalities for discrimination^21–27^. Despite these technological advances, positively identifying CTCs among blood cells is not guaranteed. Biophysical enrichment techniques are label-free, fast and typically easier-to-operate, yet they lack specificity, particularly when discriminating CTCs from nucleated blood cells^28^. Biochemical enrichment of CTCs targets certain tumor-specific membrane antigens for immunoaffinity-based discrimination offers high specificity. However, CTCs that do not express or dynamically regulate the targeted antigens are then missed, thereby making the enrichment process biased^29–33^.

A more inclusive approach for enriching CTCs from blood samples is to target the normal blood cells, which are well characterized and, more importantly, show more consistent traits among different samples or subjects. This negative enrichment approach is advantageous not only because it leads to an unbiased detection of CTCs but also produces intact tumor cells free of attached immuno-agents, eliminating potential artifacts in downstream studies^34–37^. On the other hand, negative enrichment of CTCs still primarily relies on labeling of blood samples with sample-specific amounts of magnetic beads conjugated with antibodies against membrane antigens of white blood cells (WBCs) and their depletion under a magnetic field gradient^38,39^. Depletion of white blood cells through immunocapture on functionalized device surfaces is often not practical for clinically-relevant volumes of blood because of the limited surface area available in a typical microfluidic device^40^. To increase the surface area available for the WBC immunocapture, we recently introduced an additively manufactured multi-layered microfluidic device, which still required WBCs to be prelabeled with biotin and relied on strong avidin–biotin interaction for depletion^41^.

In this work, we report a method to negatively enrich CTCs directly from unmanipulated whole blood samples collected from patients in clinically relevant volumes. We designed and manufactured a 3D microfluidic device that first captured tens of millions of non-labeled WBCs on the inner surfaces functionalized with an antibody that specifically binds to a WBC-specific membrane antigen and subsequently depleted red blood cells (RBCs) and platelets exploiting the large size contrast with the CTCs. Our label-free method can then release concentrated live CTCs from the device, making our technique compatible with immunocytochemical, molecular and functional studies downstream. To achieve this capability, in this work we (1) overcome the limited functional surface area restriction suffered by the conventional microfluidic devices with 3D microfluidic networks that cannot be fabricated using the conventional fabrication method, (2) combine leukodepletion channels and microfiltration in one device reducing the risk of losing valuable CTCs during the transportation of the sample and eliminating the need for RBC lysing that is usually required by the conventional microfluidic devices for negative enrichment and (3) utilize immuno-enhanced microfiltration to further reduce the number of WBCs in the enriched product and allow intact and label-free CTCs to be released in liquid suspension. We optimized the device design and process using spiked-cell experiments and then applied our technique on clinical samples collected from patients with pancreatic and prostate cancers to perform a pilot study using the developed technology.

## Results

### Design of the microfluidic device

**Figure 1:**
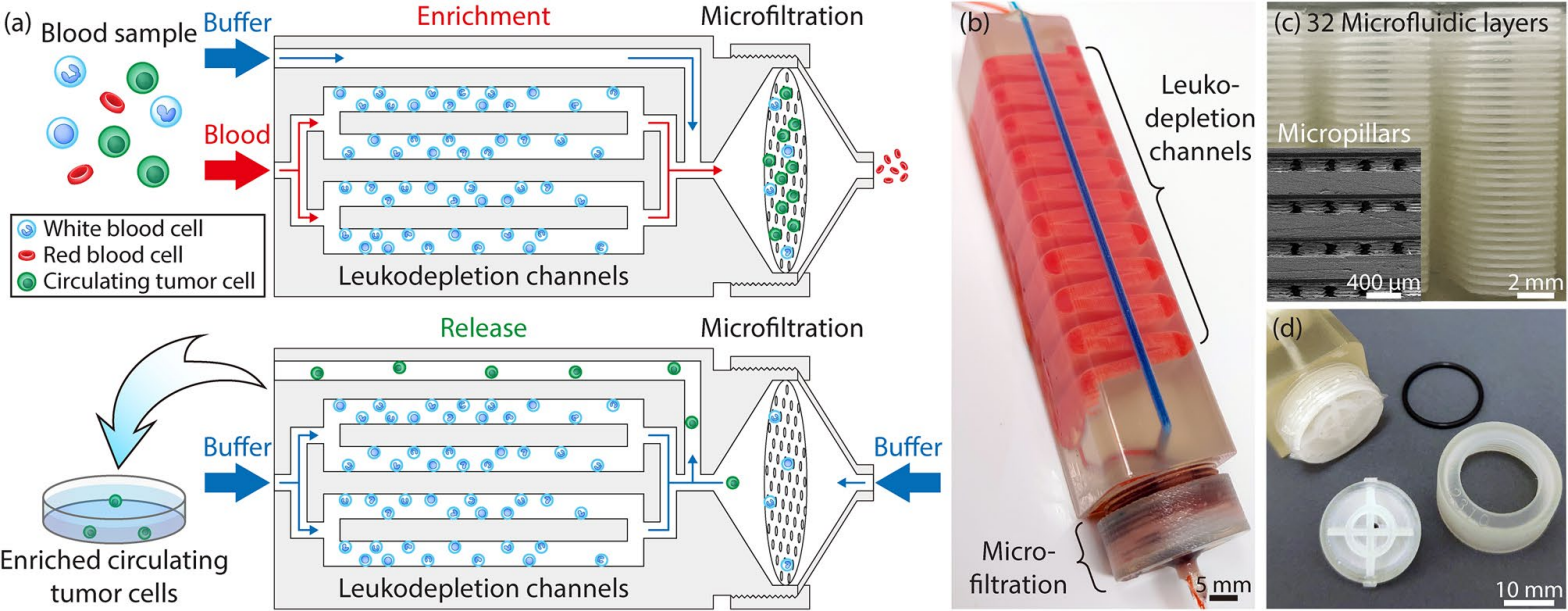
The design and operation principle of the 3D-printed microfluidic device. (**a**) Schematic illustrations of the circulating tumor cell enrichment process using the developed device. The device discriminates CTCs from normal blood cells by eliminating leukocytes through immunodepletion and anucleated blood cells through their smaller size. The enriched CTCs are then released into a petri dish under reverse flow. (**b**) A photo of the 3D-printed microfluidic device. Red and blue color dyes are injected through the inlets to visualize the multi-layered leukodepletion channels and washing buffer channel, respectively. (**c**) A close-up photo showing the stacked 32 leukodepletion channels and (inset) an SEM micrograph showing cross-section of the channels, where the micropillars within the channels can be seen. (**d**) A photo of the microfiltration stage and associated components. The filter support assembly uses an O-ring to prevent leakage during operation and a threaded lock to secure the entire assembly.

Our microfluidic device was designed to process unmanipulated whole blood and enrich the CTCs negatively. When the whole blood sample is introduced into the device, it is first uniformly distributed, via 3D bifurcating channels, into multi-layered leukodepletion channels chemically functionalized for selectively capturing WBCs in flow. The leukodepleted blood sample is then routed onto a custom-built membrane filter. Having the filter after the leukodepletion stage prevents clogging of the filter as the bulk of WBCs are already depleted at this point. The immuno-functionalized membrane filter then chemically captures residual WBCs that escaped from the leukodepletion channels and only mechanically retains the CTCs. The rest of the blood components (i.e., RBCs, platelets, and serum) can pass through the filter pores and are ejected from the waste outlet. A reverse flow is then applied from the waste outlet to release the mechanically retained CTCs from the membrane filter and recollect them in a petri dish for downstream analysis (Fig. 1a). To ensure against CTCs going into the multi-layered leukodepletion channels during the release process, a buffer was also injected from the sample inlet using a syringe pump. As a result, CTCs are directed to only unconstrained port, the dedicated release outlet, and exclusively released from there.

Structurally, the designed device consists of two main sections, a compartment with stacked layers of immuno-functionalized leukodepletion channels followed by a microfiltration compartment with an immuno-functionalized membrane filter. There are two inlets to the device: the sample inlet is coupled to the leukodepletion channels, while the buffer inlet bypasses leukodepletion channels and directly supplies to the microfiltration compartment to wash the membrane filter (Fig. 1b). While the compartments work independently, their integration on the same device prevents potential cell loss if microfluidic immunodepletion and microfiltration were to be performed separately.

The whole device measures 102 mm in length, 20.5 mm in width, and 19.2 mm in height. There are 32 parallel leukodepletion channels (Fig. 1c), each is 2.125 mm wide and 175 µm deep. The serpentine geometry provides channels with an effective length of ~ 40 cm for each layer, which was designed to increase the frequency of physical contact between the WBCs and the functionalized surface to maximize the immunocapture efficiency. The channels are vertically spaced with 300 µm gaps in between for structural integrity. Within each immunocapture channel, 200 µm-diameter micropillars were placed with a 400 µm-pitch and served to increase the functionalized surface area for higher immunodepletion efficiency while supporting vertically stacked layers across the device. Micropillars were specifically arranged to laterally shift by 10 µm from row-to-row to break the laminar flow stream and maximize the physical contact with the WBCs. With all these features taken into consideration, our device had an effective surface area of ~ 55,900 mm^2^. This surface area was projected to be sufficient in accommodating 50 million WBCs, which corresponds to the amount in a 10 mL tube of whole blood sample, on ~ 9% of the total available surface area. Having > 90% margin for the surface area allows sparse immunocapturing of cells and minimizes the effect of steric hindrance on the WBC capture.

The microfiltration compartment contains a 20 mm-diameter, 3 µm-pore, membrane filter microfabricated (“Methods”) out of polydimethylsiloxane (PDMS) along with removable 3D-printed parts to hold the filter in place (Fig. 1d). The filter itself could not be 3D-printed as with the rest of the device components due to insufficient resolution of the printer for achieving the desired pore size. 3 µm pore diameter was specifically chosen for our filter design to retain the majority of nucleated cells from whole blood, while passing much smaller RBCs and platelets. In assembling the final device, a separately fabricated membrane filter was sandwiched between two support layers with O-ring in place to prevent leakage. A threaded lock secured the whole filter compartment assembly. This particular design of the microfiltration compartment allowed direct access for inserting and removing the thin PDMS membrane filter and held the membrane stable during uncapping, which ensured against potential shear damage.

### Optimization of the antibody coverage on the device surface

To specifically capture WBCs from among other blood cells, we chemically functionalized the inner surfaces of our device against CD45, a membrane antigen commonly expressed by different WBC subpopulations^40^. To prime the device for antibody immobilization, we employed a modified version of the functionalization protocol developed by Stott et al.^42^ Prior to surface functionalization, the PDMS membrane filter was activated in oxygen plasma and then integrated into the 3D-printed microfluidic device. A series of chemical reagents (“Methods”) were introduced from the sample inlet towards the waste to coat both the stacked microfluidic channels and the membrane filter at once. Anti-CD45 antibodies were then attached to the primed device surface through avidin–biotin interaction (Fig. S1a).

To maximize the immunocapture efficiency, we next determined the optimal concentration of antibodies that would ensure complete surface coverage. In this process, antibody concentrations ranging from 0.5 to 20 µg/mL were applied on an analytical version of the 3D-printed device and the PDMS membrane filter separately to account for potential disparity due to different materials. To measure antibody coverage in our tests, immobilized mouse anti-CD45 antibodies were labeled with a secondary antibody, Alexa Fluor 488 goat anti-mouse IgG (Invitrogen, Carlsbad, CA), at a manufacturer-suggested concentration of 1 µg/mL, and the resulting surface fluorescence intensity was measured with a fluorescence microscope (Fig. S1b). Measurements on the 3D-printed and PDMS surfaces were acquired under identical exposure settings for comparison, and all signals were normalized against the measured background fluorescence. While we observed ~ 40% more fluorescence on the PDMS surface at the limit, both surfaces were found to saturate at similar points with the surface fluorescence intensity rapidly increasing only with antibody concentrations of < 10 µg/mL. Because the fluorescence signal remained virtually unchanged (< 3% difference) for concentrations > 10 µg/mL, we chose 10 µg/mL as the optimal concentration for antibody immobilization. This resulted in consuming ~ 30 µg of anti-CD45 antibody for functionalizing the whole device (immunodepletion channels and filter), which costed ~ $15.8 per test.

### Negative depletion of white blood cells with the chemically functionalized filter

**Figure 2:**
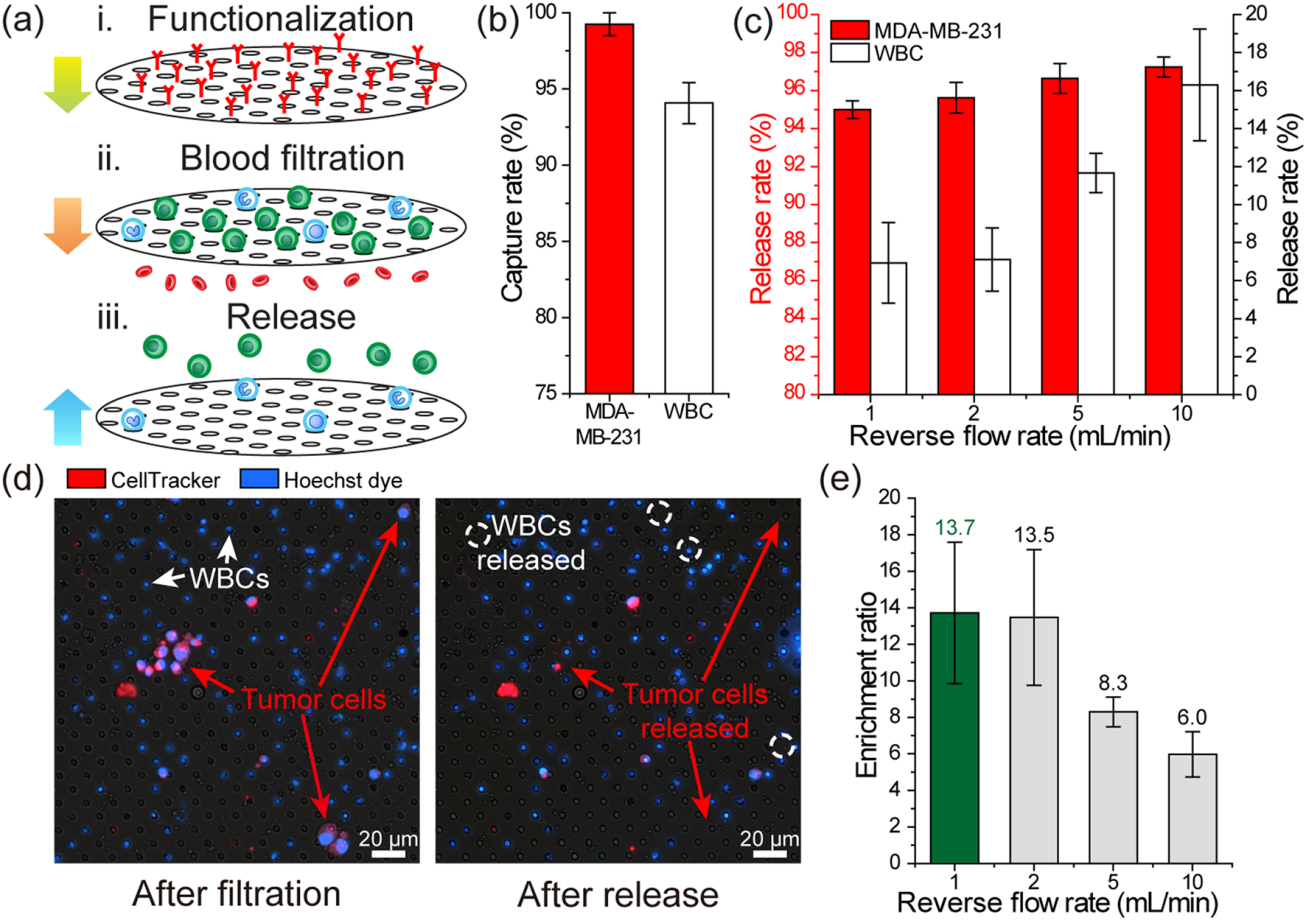
Characterization of the immuno-functionalized PDMS membrane filter for tumor cell enrichment. (**a**) A schematic showing the tumor cell enrichment process. Following the immuno-functionalization of the membrane filter, whole blood spiked with MDA-MB-231 tumor cells is driven through the filter to capture nucleated cells on the filter and discard anucleated cells. The mechanically retained tumor cells are then released from the membrane filter under reverse flow. (**b**) Measured capture rates of the MDA-MB-231 tumor cells and WBCs during filtration (n = 3). (**c**) Measured release rates of the (left axis) MDA-MB-231 tumor cells and (right axis) WBCs under different reverse flow rates (n = 3). (**d**) Fluorescence microscope images of the PDMS membrane filter (left) right after the filtration of the blood sample and (right) following the release of cells under reverse flow. The images show that the tumor cells were succesfully released from the filter while most of the immunocaptured WBCs were retained on the filter with the reverse flow. (**e**) Calculated average enrichment ratio for the spiked tumor cells as a function of different reverse flow rates.

To assess the contribution of a downstream anti-CD45-functionalized filter in depleting white blood cells, we separately tested the membrane filter outside of the device (Fig. 2a). Forward flow rates for both the sample and the buffer were set based on our earlier work that used a track-etched filter with 3 µm-diameter pores^41^. To mimic the filter operating conditions when it is in the device (i.e., a buffer mixing with whole blood during the filtration stage), we simultaneously introduced 200 µL of whole blood spiked with MDA-MB-231 human breast tumor cells, which had a measured size distribution of 5.9 µm to 19.8 µm (Fig. S2), at 2 mL/h and PBS washing buffer under 120 mbar pressure through a T-connector coupled to a commercial filter holder. We first characterized the efficiency of our microfabricated filter nucleated in capturing nucleated cells by counting fluorescently labeled tumor cells and nuclei-stained white blood cells both on the filter and in the filtrate with a fluorescence microscope. We found that our filter could retain virtually all (~ 99%) spiked tumor cells while capturing ~ 94% of the WBCs (Fig. 2b). It should be noted that the measured WBC capture rate was higher than the previously reported capture rate of 82% on a non-functionalized 3 µm track-etched membrane filter^41^. Enhanced capture efficiency was likely due to higher retention forces on WBCs due to their immunoaffinity towards the filter surface, an assessment supported by other studies on immuno-functionalized filtration^43^.

Next, we analyzed the cell population released from our filter under varying reverse flow rates to determine the optimum conditions for maximizing enrichment. For each tested flow rate, cells were subjected to 5 mL of PBS in the reverse direction and the population collected in a petri dish was imaged with a fluorescence microscope to determine the fraction of cells that were successfully released. For all reverse flow rates tested, > 95% of the spiked tumor cell population could be recovered with increasing flow rate from 1 to 10 mL/min having minimal (~ 2%) effect in enhancing the tumor cell release efficiency (Fig. 2c). While more tumor cells could be released by increasing the reverse flow rate, those potential gains were negated with the increasing number of non-specifically- or loosely-bound WBCs detaching from the filter into the product. In fact, we found the number of WBCs in the released product more than doubled when the reverse flow rate increased from 1 to 10 mL/min. Also, a direct comparison between the microscope images of pre- and post-release states of the PDMS filter confirmed the release of WBCs along with tumor cells under reverse flow (Fig. 2d). Therefore, to account for WBC contamination in the released product, we instead focused on the enrichment of tumor cells. The enrichment ratio is defined as the ratio of the tumor cells to WBCs in the final state divided by the ratio of the tumor cells to WBCs in the initial state^44^, and it can also be calculated from the ratio of the release rate of tumor cells to WBCs in our case, as shown in the equation below:

1 $$ER = \frac{{\left( {tumor \, cells/WBCs} \right)_{{final}} }}{{\left( {tumor \,cells/WBCs} \right)_{{initial}} }} = \frac{{tumor \, cells_{{final}} /tumor \, cells_{{initial}} }}{{WBCs_{{final}} /WBCs_{{initial}} }}$$

Base on the equation shown above, we calculated an enrichment ratio for each reverse flow rate (Fig. 2e). At lower reverse flow rates (1–2 mL/min), we found the functionalized filter provided more than an order of magnitude increase in CTC enrichment. The maximum enrichment ratio of ~ 13.7 × was achieved at a reverse flow rate of 1 mL/min, which we chose as the optimal flow rate for releasing from our device in subsequent studies in this work.

### Characterization of the full device with simulated blood samples

**Figure 3:**
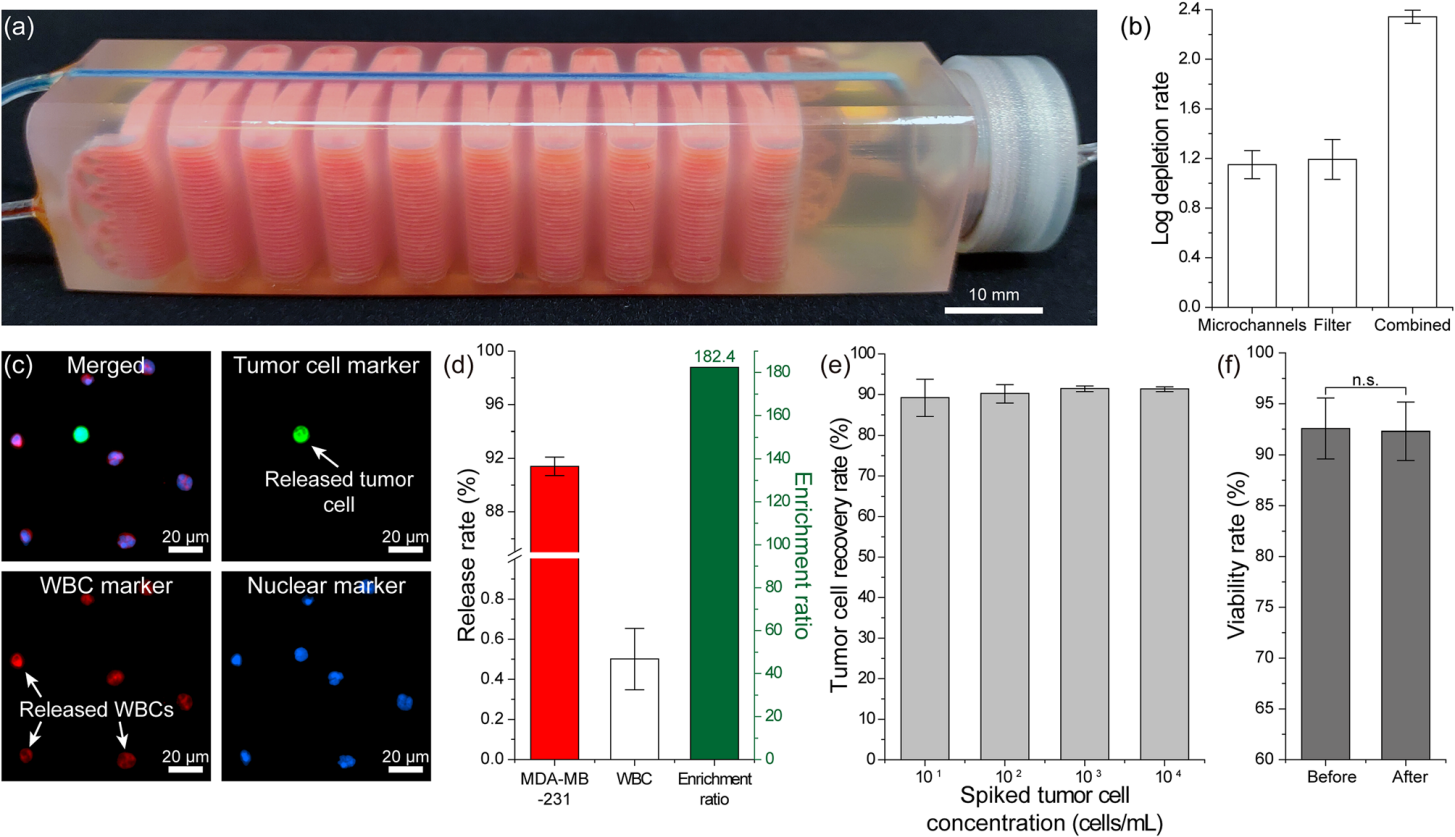
Characterization of the full device with simulated blood samples. (**a**) Photo of the final device with the PDMS membrane filter inserted in the filter compartment. The leukodepletion channels and the washing buffer channel were filled with a red and blue dye, respectively, to illustrate the device geometry. (**b**) Measured WBC log depletion rates in the leukodepletion channels, on the membrane filter, and the combined immunocapture rate for the whole device. (**c**) Fluorescence microscope images of the released cells in suspension. (**d**) Measured (left axis) release rates for the spiked tumor cells and WBCs, and (right axis) the enrichment ratio calculated based on these release rates. (**e**) Measured recovery rates of tumor cells spiked at different concentrations into blood samples. Tumor cell concentrations ranging from 10^1^ to 10^4^ cells/mL of blood were tested. A mean tumor cell recovery rate of ~ 90% was observed for all tested concentrations. The error bars represent standard deviations. (**f**) Measured tumor cell viability rates before and after processing through microfluidic device. Statistical significance was tested by performing Mann–Whitney U-tests; the difference between these two conditions was found to be not statistically significant.

To characterize the complete device, we placed the microfabricated PDMS filter into the filter compartment of our microfluidic device, chemically functionalized the device and then processed blood samples collected from healthy volunteers (Fig. 3a). We first studied the immunocapture of WBCs within the device. In these experiments, 10 mL whole blood was driven through the device at an optimized flow rate of 2 mL/h, which resulted in an average cell flow speed of ~ 100 µm/s throughout microfluidic channels and led to an efficient immunocapture of WBCs^41^. Likewise, the PBS washing buffer was pneumatically delivered to the filtration compartment at 120 mbar, which was specifically chosen to ensure efficient passage of RBCs through 3 µm-diameter pores into waste. A hematology analyzer was used to measure the initial concentration of the WBCs in the blood sample and Hoechst dye was used to stain the nuclei of the WBCs for quantification on the filter and in the filtrate after processing of the blood samples (“Methods”). By comparing the number of WBCs in the initial blood sample (on average ~ 6.3 × 10^7^ WBCs) versus the filtrate (on average ~ 2.9 × 10^5^ WBCs), we found that our device was able to achieve, on average, a ~ 2.34-log depletion of WBCs by capturing an average of ~ 6.26 × 10^7^ WBCs directly from whole blood with no labels (Fig. 3b) (Table S1). To determine the individual contributions of each section, we first subtracted the number of WBCs on the filter and in the filtrate from the initial count of WBCs in the original blood sample to obtain the number of WBCs captured by the immunodepletion channels. This number of WBCs in the immunodepletion channels was then compared with the initial count of WBCs in the original blood sample to calculate the log depletion rate for the immunodepletion channels. The log depletion rate for the membrane filter was calculated by comparing the number of WBCs passed to the filter (subtracting the number of WBCs in the original sample by the number of WBCs captured in the immunodepletion channels) with the number of WBCs found in the filtrate. We found that on average we could achieve ~ 1.15-log depletion of WBCs at immunodepletion channels while the anti-CD45 functionalized filter could provide an additional ~ 1.19-log depletion of WBCs that evaded capture at immunodepletion channels leading to a total 2.34-log WBC depletion rate from unmanipulated whole blood (Fig. 3b). Actual measured numbers of WBCs in each experiment before and after enrichment for each stage of the device are provided in Table S1.

Next, we tested our device in its ability to enrich tumor cells spiked in whole blood samples collected from healthy donors. A final tumor cell concentration of 1–5 × 10^3^ tumor cells/mL was prepared for the testing. The simulated blood samples were processed with our device, and the enriched product was released into a petri dish for analysis. We could positively identify cells in the released product for measurements since we (1) pre-labeled tumor cells with CellTracker Green before spiking, (2) immunostained WBCs against CD45 using Alexa Fluor 594 anti-human CD45 (Biolegend, San Diego, CA) on the filter prior to release. In addition, nuclei of both tumor cells and WBCs were labeled with Hoechst 33342 dye to prevent false positives (Fig. 3c). By comparing the number of tumor cells in the released product with the initial number of spiked tumor cells in the sample, we found that we could recover on average ~ 91.4% of tumor cells (Fig. 3d) where part of the cell loss was contributed by the non-specific binding of tumor cells to the leukodepletion channels (Fig. S3). To investigate if the initial tumor cell concentration had an effect on device sensitivity measurements, we also processed samples with spiked tumor cell concentrations as low as 10 tumor cells/mL of blood and found that we could still recover ~ 90% of tumor cells using our device (Fig. 3e). In agreement with experiments with non-spiked samples, we could find < 0.5% of WBCs in the released product, while anucleated blood cells (RBCs or platelets) were virtually absent. Taken together, these figures demonstrated that our device could enrich the tumor cells on average by ~ 182.4 × against the contaminating WBCs. Furthermore, we tested the viability of the enriched tumor cells in the released product (“Methods”) and found that enriched cells remained intact with no noticeable change in viability (Fig. 3f). Given the label-free enrichment process and the fact that viable tumor cells are readily available in suspension suggests the possibility of directly coupling our device with standard molecular and functional assays downstream.

### Isolation of circulating tumor cells from clinical samples

We finally used our complete 3D-printed device (with the 3 µm PDMS membrane filter inserted in the filtration compartment) to perform a pilot study and isolate CTCs from peripheral blood samples collected from patients with metastatic disease (Fig. 3a). We analyzed both prostate and pancreatic cancer samples to demonstrate the applicability of our assay with different cancer types. Blood samples for this study were collected from consenting patients at Emory University Hospital or Grady Memorial Hospital following Institutional Review Board (IRB)-approved protocols and then transported to Georgia Tech to be processed within 4 h of withdrawal. For each case, 10 mL of unmanipulated blood samples were processed with our device at 2 mL/h under optimized conditions established earlier in this paper, and the product was immunostained against tumor-specific antigens to positively identify CTCs among contaminating blood cells. Immunostaining and washing steps were performed external to the device by directly releasing the enriched population from the device onto a commercial track-etched membrane filter (Whatman plc, Maidstone, United Kingdom) with 1 µm-diameter pores to ensure complete retention. Following the fixation and permeabilization of the released cells, samples were immunostained against established prostate (Cytokeratin 8/18, vimentin, and prostate-specific antigen Kallikrein 3) or pancreatic (Cytokeratin 7/8/18, EpCAM, and vimentin) cancer markers along with WBC markers (Anti-CD45) and a nuclear stain (4′,6-diamidino-2-phenylindole (DAPI)) to positively identify enriched CTCs (“Methods”).

**Figure 4:**
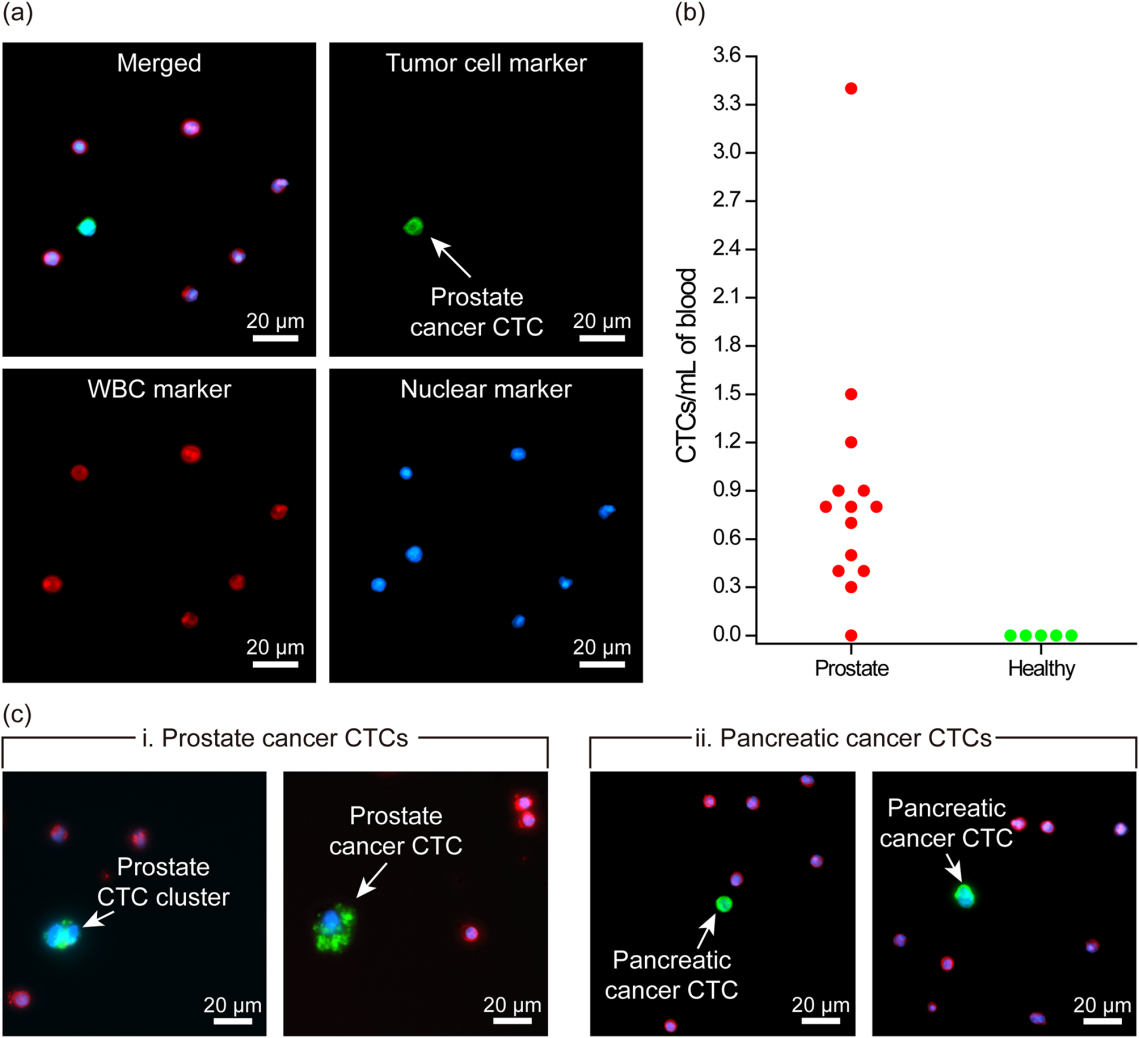
Isolation of circulating tumor cells from clinical samples. (**a**) Fluorescence microscope images of the enriched CTC from a prostate cancer patient’s blood sample. The images show the merged fluorescence image of a CTC isolated from the blood of a prostate cancer patient. Individual fluorescence channels corresponding to immunofluorescence from tumor, WBC and nuclear markers are also shown. (**b**) Measured CTC concentrations in blood samples collected from prostate cancer patients (n = 14) and healthy controls (n = 5). (**c**) Representative immunofluorescence images of patient CTCs isolated from pancreatic and prostate cancer patients’ blood samples. A two-cell CTC cluster was also found in one of the prostate samples, which demonstrates the potential use of the developed device for the enrichment of CTC-clusters.

We scored immunostained cells as CTCs only if they were positive for tumor and nuclear markers and also negative for WBC markers (Fig. 4a). In the prostate samples processed (n = 14), we observed CTC concentrations that ranged from 0 to 3.4 CTCs/mL of blood (Table S2) with a median of 0.8 CTC/mL of blood (Fig. 4b). It is worth noting that in one of the prostate cancer patient samples, we also found a two-cell cluster (Fig. 4c), demonstrating the potential of our device for isolation of CTC-clusters, which has been shown to have greater metastatic propensity than single CTCs^45,46^. In fact, relatively large features in our immunodepletion channels leave ample space for cell clusters to proceed without facing obstacles and can be seen as an advantage to protect the integrity of CTC clusters. To demonstrate the applicability of our device to process clinical samples from any metastatic cancer, we also processed a blood sample collected from a patient with pancreatic cancer and isolated CTCs at a concentration of 0.3 CTCs/mL (Fig. 4c). Finally, we processed blood samples collected from healthy volunteers (n = 5) as control and could not find CTCs in any of these samples (Fig. 4b). Taken together, these pilot study results demonstrated the clinical potential of our technology for isolating CTCs from blood samples as-withdrawn from patients with no sample preparation.

Finally, to investigate the potential loss of CTCs in clinical samples, we examined the waste from three randomly selected patient samples. To look for CTCs in those waste samples, we lysed the red blood cells, fixed the remaining nucleated cells and then retain the fixed cells on a 1 µm diameter-pore membrane filter for immunostaining (“Methods”). Imaging of immunolabeled samples with a fluorescence microscope could not identify a CTC in those samples, demonstrating the validity of our results from the characterization of our technology with simulated blood samples.

## Discussion

In this paper, we introduced a CTC enrichment technology that utilizes the design flexibility provided by the 3D-printing technology to create a microfluidic device that combines multi-parallel immunodepletion channels and immune-enhanced microfiltration in series to achieve negative enrichment of CTCs directly from clinically relevant volumes of unmanipulated whole blood samples. The integration of microfiltration after the immunodepletion channels that remove most of the WBCs from the blood sample allows us to take advantage of the significant size contrast between anucleated and nucleated cells in the blood and use a filter membrane with a much smaller pore-size (3 µm) for the separation as compared to the conventional microfiltration methods that are limited to the use of 5–10 µm pore size membrane filter to prevent clogging of the filter by the large number of WBCs^47–49^. While we cannot exclude the possibility of losing very small CTCs during the filtration stage, the majority of CTCs are expected to be retained with a 3 µm-diameter-pore membrane filter given the larger sizes of CTCs reported in the literature and high CTC recovery rates reported by conventional filtration methods even with larger pore size^50,51^.

We designed and optimized our device to process a 10 mL tube of whole blood sample as it represents a typical sample size collected in blood-based clinical investigations. However, the device size can be increased to process larger volumes of blood samples to enhance the likelihood of detecting rare CTCs. A scaled version of our device can accommodate more WBCs within, and therefore, isolate CTCs with no penalty on detection sensitivity due to immunocapture surface saturation. The experimental setup used in this work readily allows 3D printing of devices with > 10 × the current volume, while the centrifugation step would require a custom bucket large enough to accommodate the scaled device.

With our device, we performed a pilot study and processed clinical blood samples from both prostate and pancreatic cancer patients with no sample manipulation. The measured concentrations of CTCs among 14 prostate cancer samples were as high as 3.4 CTCs/mL with a median concentration of 0.8 CTCs/mL, which is in agreement with previous reports on the prevalence of CTCs in prostate cancer^52^. As for the pancreatic patient sample that we processed, we found 0.3 CTC/mL of blood, similar to the CTC concentration reported by Gao et al. in the pancreatic cancer patient samples^53^. Furthermore, the low CTC concentration in the blood sample can also be due to treatment. Rivera-Baez et al. reported that the number of CTCs per milliliter of blood greatly decreases after chemotherapy^54^. These demonstrated that our device is capable of recovering CTCs effectively. Measured concentrations of CTCs are in agreement with previously reported results from other CTC assays on prostate cancer patients especially considering our cohort was formed by metastatic patients undergoing therapy (Table S2)^55^.

Lastly, although the depletion of ~ 99.6% of WBCs directly from unmanipulated whole blood samples is a technological achievement, it still leaves ~ 10^5^ contaminating WBCs in the product when a 10 mL blood sample is processed. For this study, the low purity of the product was not at the level to obscure isolated CTCs and did not interfere with immunofluorescence measurements. However, contaminating WBCs would introduce noise in molecular assays such as DNA or RNA sequencing and can lead to confounding results^56,57^. Nevertheless, the purity of CTCs in the final product can potentially be increased for such assays. One approach would be to micromanipulate CTCs released from the device and transfer them to another container, virtually eliminating all contamination. Another approach would be to improve the depletion rate of WBCs (1) by functionalizing the device with a cocktail of WBC-specific antibodies and (2) by creating smaller channels with finer structures, either of which would enhance the WBC immunocapture efficiency, and therefore, improve the purity of the CTCs in the final product.

## Conclusion

The ability to reliably harvest tumor cells from peripheral blood of cancer patients in a practical manner will not only transform how cancers are diagnosed and managed but also impact the basic research on metastasis and drug development. In this work, we reported a label-free blood test that relies on a combination of chemical and physical markers to specifically identify CTCs with no sample manipulation whatsoever. Compared to previously reported label-free CTC assays, our technology is more specific than ones that solely rely on biophysical parameters for CTC identification and can actually process clinically relevant volumes of blood samples with no lysis of RBCs unlike microfluidic devices that aimed to deplete WBCs with immunocapture. In that respect, our technology combines the practicality of biophysical methods with the specificity of biochemical CTC isolation. The fact that the enrichment process does not rely on tumor-specific markers makes our assay applicable virtually on all cancers with solid tumors. Furthermore, utilizing a commercially available 3D-printer to create the bulk of the fully-functional CTC assay suggests the intriguing possibility of on-demand, decentralized manufacturing of these tests at homes/offices especially considering both the additive manufacturing becoming widely accessible for personal use and the fast-paced innovation in instrumentation and materials.

## Methods

### 3D-printing of the device

A 3D model of the microfluidic device was drawn with a computer-aided design (CAD) software (SolidWorks Corp., Waltham, MA). The device was fabricated with a commercially available 3D printer (ProJet 3510 HD from 3D Systems, Rock Hill, SC) using VisiJet M3-X plastic material (3D Systems, Rock Hill, SC), which was chosen due to both its high modulus of elasticity (2.168 GPa) for rigidity and its optical transparency to facilitate visual investigation of the device in operation. In addition, the material had a high heat distortion temperature (88 °C) which prevented damage to the microfeatures of the device during post-processing. After printing, the device was centrifuged in a 50 mL conical as immersed in mineral oil (Durvet, Blue Springs, MO) to discharge the wax support material from microchannels. In this process, the tube was placed in a 65 °C oven for 2 h to ensure that all of the wax support materials were liquefied before centrifuging at 500×*g* for 1 h. A heat gun was used to supply hot air into the centrifuge during the process to maintain the temperature at 65 °C. The device was later centrifuged in soapy water (P&G, Cincinnati, OH) and deionized (DI) water, respectively, to remove the residual mineral oil and soapy water from the microchannels. With uniform forces across the device, centrifugation was observed to efficiently remove wax from the microchannels at the end of this process.

### Microfabrication of the membrane filter

We fabricated the membrane filter out of PDMA using spin-cast soft lithography. First, we manufactured the mold with an array of micropillars. A 4-inch silicon wafer (UniversityWafer, Inc., Boston, MA) was first spin-coated with SU-8 2000 series photoresist (MicroChem, Westborough, MA) at 4000 rpm to create a 25 µm-thick photoresist film. The photoresist film was patterned with conventional photolithography to form a 30 mm × 30 mm 3 µm micropillar array with a 25% duty cycle, which was projected to produce a membrane filter with a pore density of 6950 pores/mm^2^ (Fig. S4a). After ensuring the mold is defect-free with a scanning electron microscope (SEM), a thin film of NR9-1500PY photoresist (Futurrex, Inc., Franklin, NJ) was spin-coated onto the mold as a sacrificial layer to facilitate demolding. In this process, to achieve a final thickness of < 1 µm, the wafer was first spun at 600 rpm to evenly spread the photoresist and then at 4000 rpm. Next, a mixture (10:1) of PDMS elastomer Sylgard 184 and crosslinker (Dow Corning, Auburn, MI) was degassed in a desiccator and spin-coated on the sacrificial photoresist film at 3000 rpm, which produced a PDMS layer thick enough to fully cover all micropillars. We then uniformly etched the excess PDMS on micropillars to achieve through-holes by spinning 200 µL of hexane at 1000 rpm across the whole wafer and cured on a hot plate at 120 °C for 10 min^58^. At this point, micropillars were observed to protrude from the PDMS film as intended (Fig. S4b). Next, the PDMS membrane filter was released from the mold by etching the sacrificial photoresist in acetone (Fig. S4c). The hydrophobicity of the PDMS membrane allowed it to float atop of the acetone after release^59^, which we utilized to transfer the thin membrane filter onto the 3D-printed device (Fig. S4d). Scanning electron micrograph of the final PDMS membrane was observed to be free of defects and uniformly sized and spaced with through holes throughout the entire functional area.

### Immuno-functionalization of the device

To functionalize the microfluidic channels and membrane filter for immunocapturing WBCs, we first wetted the device with 200 proof ethanol (Thermo Fisher Scientific, Waltham, MA), and then incubated the device in 3-mercaptopropyl-trimethoxysilane (MPTMS) (Gelest, Morrisville, PA) in ethanol (4% v/v) for 1 h at room temperature. Next, a 100 mg/mL stock solution of N-y-maleimidobutyryloxy succinimide ester (GMBS) (Pierce Biotechnology, Rockford, IL) in dimethyl sulfoxide (DMSO) (Pierce Biotechnology, Rockford, IL) was prepared and further diluted in ethanol at 0.28% v/v ratio. Following an ethanol rinse, the device was filled with the prepared GMBS solution in ethanol and incubated for 30 min at room temperature. The device was then sequentially rinsed with ethanol and phosphate-buffered saline (PBS) (Corning Inc., Corning, NY). The device was then incubated with a 10 µg/mL-solution of NeutrAvidin (Pierce Biotechnology, Rockford, IL) in PBS for 1 h at room temperature. Later, biotinylated mouse anti-human CD45 antibodies (SouthernBiotech, Birmingham, AL) mixed in PBS was introduced to the device and incubated for 1 h at room temperature. Following a PBS wash, the device was finally incubated with SuperBlock T20 (PBS) Blocking Buffer (Thermo Fisher Scientific, Waltham, MA) for 1 h to block all non-specific binding sites completing the functionalization process (Fig. S1a). Taken together, the total incubation time for priming the devices was 4.5 h.

### Processing blood samples on the device

To test and optimize our microfluidic device, we processed fresh whole blood samples collected from consenting healthy volunteers. All blood samples were processed on the device at a rate of 2 mL/h. At the end of the process, the device was washed in PBS for 1 h and the product was released in ~ 5 min into a petri dish. Taken together, a 10 mL whole blood sample took ~ 6 h 5 min to complete. All research and experimental protocols were approved by the Institutional Review Board (IRB) at Georgia Institute of Technology and studies were performed in accordance with the relevant guidelines and ethical regulations of the IRB-approved protocol. Written informed consent was obtained from all volunteers for their participation in this study. Blood samples were withdrawn into tubes containing anti-coagulant ethylenediaminetetraacetic acid (EDTA) (BD, Franklin Lakes, NJ) and stored on a rocker at room temperature to prevent settling until they are processed within 4 h of the collection. Complete blood count (CBC) was performed on all samples with a hematology analyzer (Abbott CELL-DYN Emerald) to measure the initial concentration of the WBCs. The number of WBCs captured on the filter was quantified by immunostaining nuclei of WBCs with Hoechst dye and subsequently examining the filter under a fluorescence microscope (Nikon Eclipse Ti-E). WBC counts were acquired from multiple locations (> 10) to determine the average number of WBCs per unit area. The total number of WBCs on the whole filter was then estimated based on the measured average concentration. The total number of WBCs in the waste was estimated by measuring the concentration of WBCs in the waste with a Nageotte Hemacytometer. For quantification, the nuclei of WBCs were also stained with the Hoechst dye. The total number of WBCs in the waste was calculated from the average measured concentration of WBCs and the total volume of the waste.

To investigate device performance for tumor cell enrichment, we processed simulated samples prepared by spiking a known amount of the tumor cells into whole blood samples collected from healthy donors. Human breast cancer cells MDA-MB-231 (ATCC HTB-26) were cultured in Dulbecco’s Modified Eagle's medium (Corning Inc., Corning, NY) supplemented with 10% fetal bovine serum (FBS) (Seradigm, Radnor, PA) in an incubator at 37 °C and 5% CO_2_. Once reached 80% confluency, cells were suspended via trypsinization and labeled with CellTracker Orange or Green dyes for identification with a fluorescence microscope. Fluorescently labeled tumor cells were then spiked in whole blood samples to achieve final concentrations of 1–5 × 10^3^ tumor cells/mL. The viability of enriched tumor cells was measured with a live/dead cell assay (ab115347) (Abcam, Cambridge, UK) according to the manufacturer-suggested protocol.

### Immunofluorescence staining of patient CTCs

Blood samples from prostate and pancreatic cancer patients were obtained under the IRB-approved protocols at Grady Memorial Hospital and Emory University Hospital, respectively, according to the relevant guidelines and regulations. Informed consent was obtained from all patients according to the IRB-approved protocols. To immunostain the CTCs enriched from cancer patient blood samples, cells released from our device were first fixed in 4% paraformaldehyde (PFA) (Electron Microscopy Sciences, Hatfield, PA) and permeabilized with 1% Nonidet-P40 (Thermo Fisher Scientific, Waltham, MA). Prostate cancer CTCs were labeled with primary antibodies against Cytokeratin 8/18 (Invitrogen, Carlsbad, CA), vimentin (Invitrogen, Carlsbad, CA), and prostate-specific antigen Kallikrein 3 (PSA/KLK3) (Cell Signaling, Danvers, MA)^52,60,61^. Pancreatic cancer CTCs were immunostained against Cytokeratin 7/8/18 (Invitrogen, Carlsbad, CA), EpCAM (Invitrogen, Carlsbad, CA), and vimentin^62–64^. For both cancer types, a matching secondary antibody with Alexa Fluor 488 (Invitrogen, Carlsbad, CA) was then applied to generate the fluorescence signal from immunolabeled CTCs. Contaminating WBCs were labeled with Anti-CD45 (BD Biosciences, San Jose, CA) primary antibody followed by Alexa Fluor 594 (Invitrogen, Carlsbad, CA). Finally, 4′,6-diamidino-2-phenylindole (DAPI) (Invitrogen, Carlsbad, CA) was used to stain the nuclei of all nucleated cells.

## Supplementary Information

Supplementary Information.
