# Supplementary material for: Negative enrichment of circulating tumor cells from unmanipulated whole blood with a 3D printed device
Source: Sci Rep. 2021 Oct 18;11:20583. doi: 10.1038/s41598-021-99951-0 (PMC8523721; doi:10.1038/s41598-021-99951-0)
Supplement: Supplementary file 1 — Supplementary Information. [file 41598_2021_99951_MOESM1_ESM.pdf]

Supporting Information

Negative Enrichment of Circulating Tumor Cells from Unmanipulated Whole Blood with a 3D Printed Device

Chia-Heng Chu, Ruxiu Liu, Tevhide Ozkaya-Ahmadov, Brandi E. Swain, Mert Boya, Bassel El-Rayes, Mehmet Akce, Mehmet Asim Bilen, Omer Kucuk, and A. Fatih Sarioglu*

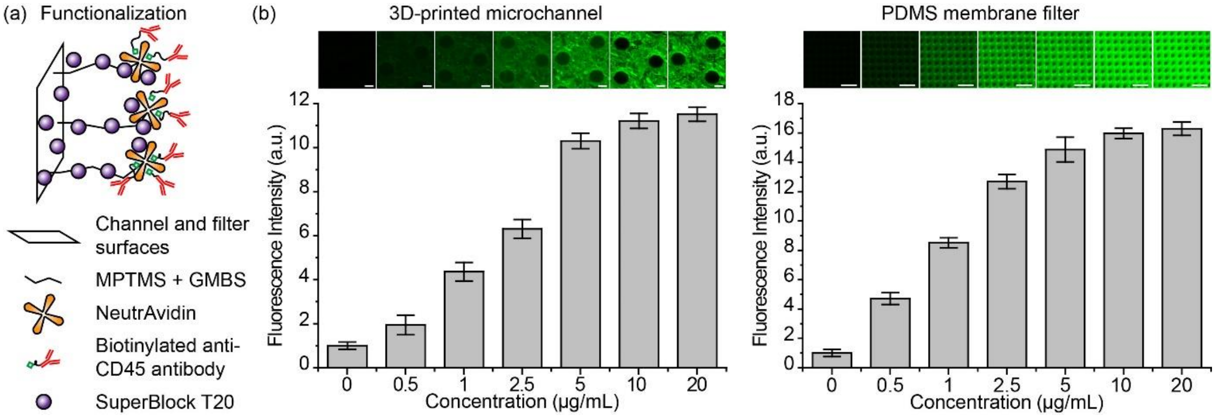

Figure S1. Functionalization of the 3D-printed microchannels and PDMS membrane filter surfaces and optimization of the antibody concentration. (a) A schematic showing the specific chemistry used to immobilize anti-CD45 antibody immobilization on the device surface. (b) Measured normalized fluorescence signal intensity from anti-CD45 on (left) the 3D-printed microchannels and (right) the PDMS filter as a function of the antibody concentration used for incubation. Fluorescence intensity for each antibody concentration was normalized against the measured background intensities of each material. The scale bars represent 500 μm for images of the 3D-printed microchannel and 20 μm for images of the PDMS membrane filter.

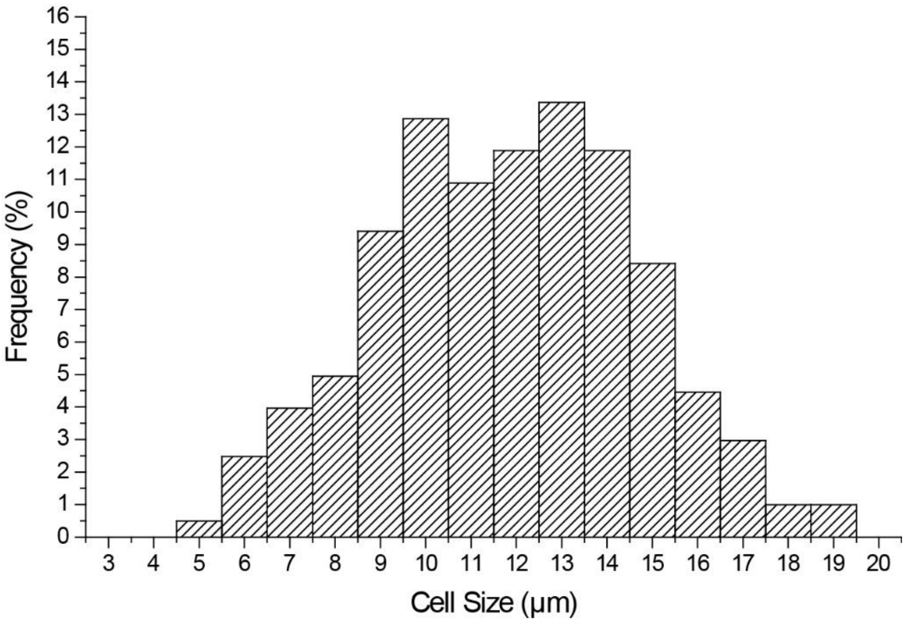

Figure S2. Measured cell size distribution of MDA-MB-231 tumor cells (n=202) sampled from the cell culture used in the experiments. The histogram shows the cell size varies from 5.9 μm to 19.8 μm.

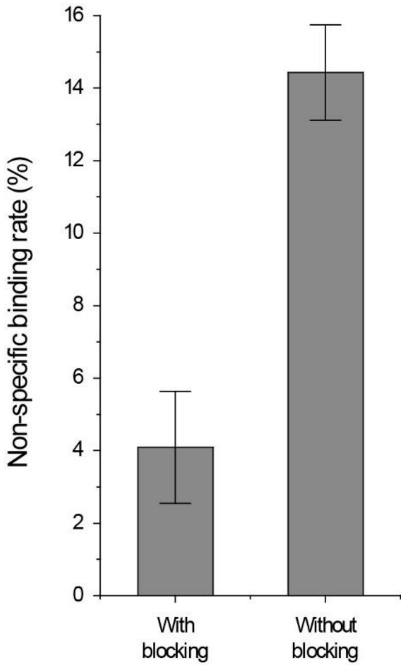

Figure S3. Non-specific binding of the MDA-MB-231 tumor cells in the microchannels. After functionalizing the leukodepletion channels, non-specific binding of the MDA-MB-231 tumor cells was tested for devices with and without the Superblock™ T20 Blocking Buffer. The results showed that the blocking buffer effectively reduced the non-specific binding of the tumor cells in the functionalized channels.

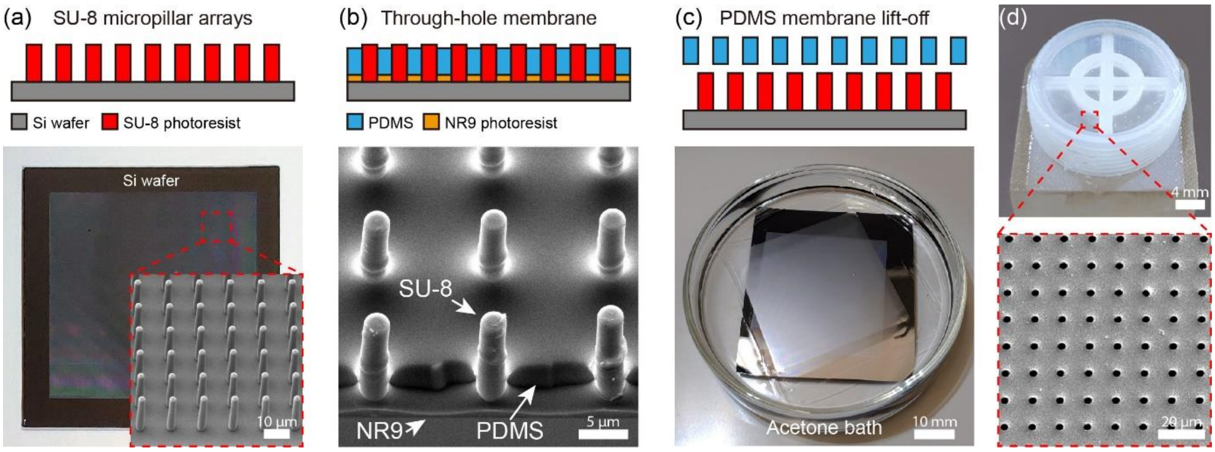

Figure S4. Microfabrication of the 20 mm-diameter 3 µm pore size PDMS membrane filter. (a) (top) Fabrication illustration of the 3 µm SU-8 micropillar arrays on a silicon substrate using the conventional photolithography technique. (bottom) Image of the fabricated SU-8 mold on a silicon substrate with (inset) scanning electron micrograph of the SU-8 micropillar arrays. (b) (top) Fabrication illustration of the through-hole PDMS membrane on the SU-8 mold through a sequential spin-coating of NR9 sacrificial photoresist, PDMS, and hexane, respectively. (bottom) Scanning electron micrograph showing the successful micromolding of the PDMS for creating through-hole membrane. (c) (top) Fabrication illustration of the PDMS membrane filter demolded through the etching of the NR9 sacrificial photoresist. (bottom) Picture of the released PDMS membrane filter from the SU-8 mold in an acetone bath. (d) (top) Photo of the PDMS membrane filter in the filtration compartment of the 3D-printed device with (bottom) an SEM image of the completed PDMS membrane filter.

Table S1. Number of WBCs Captured from 10 mL Whole Blood Samples at Different Stages of the 3D Printed Microfluidic Device.

	**Measured Number of WBCs**			
**Sample #**	**In the Original Sample (WBCs)**	**Captured by the Leukodepletion Channels (WBCs)**	**Captured by the Filter (WBCs)**	**In the Filtrate (WBCs)**
**1**	6.480×10^7	6.138×10^7	3.084×10^6	3.370×10^5
**2**	7.140×10^7	6.521×10^7	5.876×10^6	3.142×10^5
**3**	5.250×10^7	4.843×10^7	3.854×10^6	2.153×10^5
**Avg.**	6.290×10^7	5.834×10^7	4.271×10^6	2.888×10^5

Table S2. Number of CTCs detected in control, prostate, and pancreatic patients' samples.

Patient Type	Patient ID	Number of CTCs	CTC Density (CTCs/mL)	Metastatic (Y/N)	Treatment (Y/N)	Stage of Disease (I/II/III/IV)
Prostate	Pro_001	8	0.8	Y	Y	IV
	Pro_002	12	1.2	Y	Y	IV
	Pro_003	0	0.0	Y	Y	IV
	Pro_004	34	3.4	Y	Y	IV
	Pro_005	15	1.5	Y	Y	IV
	Pro_006	5	0.5	Y	Y	IV
	Pro_007	7	0.7	Y	Y	IV
	Pro_008	4	0.4	Y	Y	IV
	Pro_009	8	0.8	Y	Y	IV
	Pro_010	8	0.8	Y	Y	IV
	Pro_011	9	0.9	Y	Y	IV
	Pro_012	3	0.3	Y	Y	IV
	Pro_013	4	0.4	Y	Y	IV
	Pro_014	9	0.9	Y	Y	IV
Pancreatic	Pan_001	3	0.3	Y	Y	IV
Healthy Control	Hea_001	0	0.0	NA	NA	NA
	Hea_002	0	0.0	NA	NA	NA
	Hea_003	0	0.0	NA	NA	NA
	Hea_004	0	0.0	NA	NA	NA
	Hea_005	0	0.0	NA	NA	NA
